# The effect of arginine glutamate on the stability of monoclonal antibodies in solution

**DOI:** 10.1016/j.ijpharm.2014.06.053

**Published:** 2014-10-01

**Authors:** Priscilla Kheddo, Malgorzata Tracka, Jonathan Armer, Rebecca J. Dearman, Shahid Uddin, Christopher F. van der Walle, Alexander P. Golovanov

**Affiliations:** a Manchester Institute of Biotechnology, University of Manchester, Manchester M1 7DN, UK; b Faculty of Life Sciences, University of Manchester, Manchester M13 9PL, UK; c MedImmune Ltd., Granta Park, Cambridge CB21 6GH, UK

**Keywords:** Protein aggregation, Excipients, mAbs formulation, Physical characterization, Physical stability

## Abstract

Finding excipients which mitigate protein self-association and aggregation is an important task during formulation. Here, the effect of an equimolar mixture of l-Arg and l-Glu (Arg·Glu) on colloidal and conformational stability of four monoclonal antibodies (mAb1–mAb4) at different pH is explored, with the temperatures of the on-set of aggregation (*T*_agg_) and unfolding (*T*_m1_) measured by static light scattering and intrinsic fluorescence, respectively. Arg·Glu increased the *T*_agg_ of all four mAbs in concentration-dependent manner, especially as pH increased to neutral. Arg·Glu also increased *T*_m1_ of the least thermally stable mAb3, but without similar direct effect on the *T*_m1_ of other mAbs. Raising pH itself from 5 to 7 increased *T*_m1_ for all four mAbs. Selected mAb formulations were assessed under accelerated stability conditions for the monomer fraction remaining in solution after storage. The aggregation of mAb3 was suppressed to a greater extent by Arg·Glu than by Arg·HCl. Furthermore, Arg·Glu suppressed the aggregation of mAb1 at neutral pH such that the fraction monomer was near to that at the more typical formulation pH of 5.5. We conclude that Arg·Glu can suppress mAb aggregation with increasing temperature/pH and, importantly, under accelerated stability conditions at weakly acidic to neutral pH.

## Graphical abstract

**Figure:**
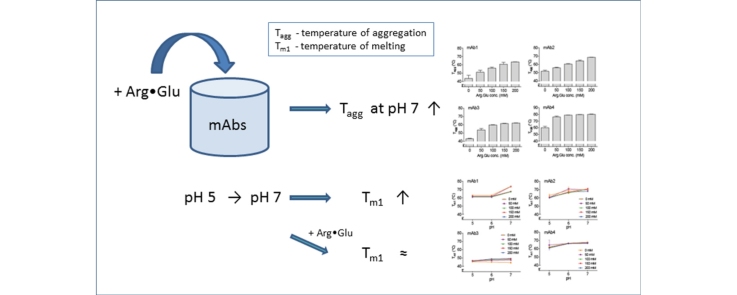

## 1 Introduction

Monoclonal antibodies (mAbs) are promising therapeutic drugs for the treatment of a wide range of diseases (Chan and Carter, 2010, Leavy, 2010, Smith and Clatworthy, 2010, Weiner et al., 2010). Their success is due to various properties including their high binding specificity and affinity, robust manufacturing processes, and the availability of humanized forms that attenuate immunogenic responses (Beck et al., 2010). However, mAb self-association and aggregation is sometimes observed during formulation at high concentrations (>100 mg/ml), and also with environmental stresses such as shaking, changes in solution pH, freeze–thaw and elevated temperatures (Banga, 2006, Chaudhuri et al., 2014, Cromwell et al., 2006, Wu et al., 2014). Hence, optimization of a mAb formulation by controlling buffer, ionic strength and pH as well as the addition of excipients is crucial in minimizing the extent of aggregation (Aboel Dahab and El-Hag, 2014, Goldberg et al., 2011, He et al., 2011, Saito et al., 2013, Sule et al., 2012).

All globular proteins, including mAbs are known to be susceptible to aggregate formation. The term ‘protein aggregation’ can be defined in a broad sense as any pathway forming protein assemblies, or aggregates (Mahler et al., 2009). Aggregation may result from the reversible self-association of the native protein, or irreversible formation of non-native assemblies following the partial or complete unfolding. (Aggregation through changes in post-translational modification and chemical degradation will not be considered here.) Self-association involving native protein–protein interaction may occur through complementary surface effects, attractive electrostatic or short range attractive forces (He et al., 2011, Liu et al., 2005). Such intermolecular self-association of proteins is related to the colloidal stability, which can be assessed by, for example, measuring temperature at which light becomes scattered by protein aggregates appearing. Aggregation involving partially unfolded protein may occur via exposed hydrophobic patches, generating non-native assemblies, and is related to the conformational stability of a protein (Arzenšek et al., 2012, Goldberg et al., 2011, Pace et al., 1996, Shi et al., 2013). Conformational stability can be assessed by measuring the temperature of protein melting transition. Ideally, increasing both the colloidal and conformational stability would be beneficial for creating a stable formulation, however in practice optimizing one of these parameters may compromise the other.

When considering approaches to choosing mAbs formulation for the best stability, the correct selection of buffer, pH and excipient(s) is essential. The solution pH can have profound effects on protein structure, stability and biological activity (Kopec and Schneider, 2011, Thakkar et al., 2012). In the context of formulation, pH is optimized to minimize physical and chemical degradation pathways (Cromwell et al., 2006, Gokarn et al., 2008). Generally, mAbs with a pI around 8–9 are formulated in mildly acidic buffer, avoiding for example deamidation and aggregation sometimes occurring in mildly alkaline buffer. These conditions however are not necessarily the best for the optimal conformational stability. Another difficulty arises from the limited choice of excipients available for formulation of pharmaceutical mAbs: only those listed as Generally Recognized as Safe (GRAS) by the regulatory bodies are used in practice (Ogaji et al., 2011, Pifferi and Restani, 2003). Presently, the process of formulation (i.e. choosing the best solution conditions and excipients) takes into account the protein's physiochemical properties and may also involve high-throughput screening (Li et al., 2011). Although there is no ‘universal excipient’ able to stabilize all the proteins, discovering a combination of excipients which would be applicable for a wider range of proteins is highly desirable.

An equimolar combination of the free amino acids l-arginine and l-glutamic acid (Arg·Glu) has been previously suggested (Golovanov et al., 2004) as a way to increase the solubility limit and long-term stability of several diverse proteins prone to aggregation; since then the method has been widely adopted in protein structural and functional studies (Blobel et al., 2007, Blobel et al., 2011, Hautbergue and Golovanov, 2008, Valente et al., 2005, Vedadi et al., 2006). l-Arginine itself (normally used in a form of a hydrochloride salt, Arg·HCl, to bring its solution pH down to neutral) is a widely known additive which often is used to assist protein refolding and reduce aggregation and solution viscosity (Arakawa et al., 2007, Chen et al., 2008, Das et al., 2007, Fukuda et al., 2014, Liu et al., 2005, Schneider et al., 2011, Vagenende et al., 2013). However a number of studies established that on a per-mole basis, Arg·Glu is much more effective at reducing intermolecular attractions and aggregation than l-Arg (Golovanov et al., 2004, Valente et al., 2005, Vedadi et al., 2006). The mechanism of Arg·Glu effect has been investigated using experimental (Blobel et al., 2011) and in silico methods (Shukla and Trout, 2011), which explained the synergy of the action of l-Arg combination with l-Glu. The significant effect of Arg·Glu on preventing protein aggregation is observed already at 50 mM (Golovanov et al., 2004), with an in silico study suggesting that an “optimum” concentration for an anti-aggregation effect may exist in the range of 100–200 mM, at least for the protein used for the simulations (Shukla and Trout, 2011). Recently, the stabilizing effect of high concentrations (up to 0.5 M) of Arg·Glu versus Arg·HCl on a selected IgG1 has been explored which suggested that having l-Glu (or l-Asp) as counter-ions counteracts the potentially disadvantageous destabilizing effects of l-Arg (Fukuda et al., 2014). Despite the growing popularity of using Arg·Glu as excipients for increasing protein solubility and preventing protein aggregation, to our knowledge, the systematic studies of their utility for diverse mAbs in the context of formulation as pharmaceuticals has not yet been reported.

Here we used high-throughput analysis to screen the aggregation propensity and thermal stability of mAbs in a variety of conditions. We first investigated the concentration- and pH-dependent effect of Arg·Glu (in the pharmaceutically-acceptable osmolality range) on the temperatures of the on-set of aggregation (*T*_agg_) and first melting transition (*T*_m1_) of four IgG1 mAbs as assessed by static light scattering (SLS) and intrinsic fluorescence, respectively. The effect of buffer type and solution pH on the stability of selected mAb formulations was then explored under accelerated stability conditions (storage at elevated temperature for a number of weeks), analysed for the fraction monomer by size exclusion high pressure liquid chromatography (SE-HPLC). The results suggest that using Arg·Glu as excipient at concentrations <200 mM can reduce temperature-induced aggregation of mAbs especially at pH approaching neutral, where the inherent conformational stability of mAbs is theoretically higher.

## 2 Materials and methods

### 2.1 Monoclonal antibodies and sample preparation

The four different mAbs (IgG1 with MWs from ∼145 to 148 kDa) tested here were kindly provided by MedImmune. The isoelectric points (pI) of mAb1, mAb2, mAb3 and mAb4 are 7.9–8.3, 8.44, 8.56 and 8.53, respectively; all values were measured experimentally except for that of mAb2 which was calculated. For SLS and intrinsic fluorescence measurements, the mAbs were diluted to 1 mg/mL in 10 mM citrate–phosphate (C–P) buffer (pH 5–7). These solutions were supplemented with varying concentrations of Arg·Glu (50–200 mM) as required, using prepared 1 M stock solution (Golovanov et al., 2004) containing equimolar mixture of the free amino acids l-Arg (Analytical grade, Sigma–Aldrich) and l-Glu (USP-FCC grade, J.T. Baker) in MilliQ water (18.2 MΩ cm), with pH adjusted where necessary. For preparation of buffers containing Arg·HCl, the hydrochloride salt of l-Arg was used (USP-FCC grade, J.T. Baker). The mAbs were diluted to 0.5 mg/mL for absorption measurements at 280 nm. For SE-HPLC, mAbs were diluted to 10 mg/mL in the appropriate buffer.

### 2.2 Determining solution osmolality

The osmolality of Arg·Glu solutions in the presence and absence of a mAb2 was measured using an Osmomat 030-D Cryoscopic Osmometer (Gonotec GmbH, Berlin, Germany). Measurement results are shown in Supplementary Information, Fig. S1. Arg·Glu concentrations of 5, 10, 25, 50, 100, 150 and 200 mM were prepared from a 1 M stock in MilliQ water, and also at concentrations of 50, 150 and 200 mM in 10 mM C–P buffer, pH 6.0. For solutions containing protein, mAb2 was buffer exchanged using overnight dialysis into 10 mM C–P buffer, pH 6.0, containing Arg·Glu concentrations of 50, 150 and 200 mM. The protein concentration was adjusted by dilution with the appropriate Arg·Glu solution to 30 mg/ml; concentrations were verified in triplicate using a Nano-Drop 2000 (Thermoscientific, Stafford House, Hertfordshire), by measuring optical absorption at 280 nm.

### 2.3 Static light scattering and intrinsic fluorescence

SLS and intrinsic fluorescence measurements were conducted simultaneously using an Optim 2 (Avacta, Thorp Arch Estate, Wetherby). Data was processed using the standard Optim analysis software provided (Avacta, 2013a), as per manufacturer's recommendations (Avacta, 2013b). Briefly, the SLS at 266 nm was used as an indicator for “colloidal stability”, reporting the onset of aggregation temperature (*T*_agg_), which can be defined as the temperature at which the measured scatter reaches a threshold that is approximately 10% of its maximum value (for typical trace, see Supplementary Information, Fig. S2). The changes in the SLS signal represented changes in the weight average molecular mass observed due to protein aggregation. The conformational stability was assessed by measuring the temperature of the on-set of melting, namely the mid-point temperature of the first unfolding transition, *T*_m1_ (see Supplementary Information, Fig. S3), monitored by an intrinsic fluorescence intensity ratio (350/330 nm) which is sensitive to the tryptophan exposure as protein unfolds (Avacta, 2013b). The samples were heated from 20 to 90 °C using 1 °C increments, with an equilibration time of 60 s before each point measurement. The sample measurements were made in triplicate and statistical analysis made using GraphPad Prism v6.

### 2.4 Controlled temperature storage stability studies

Accelerated stability studies were set up for mAb1, mAb3 and mAb4 (there was insufficient resource for mAb2) at concentrations between 30–50 mg/mL in 10 mM C–P buffer. Two formulations were prepared for each mAb in order to compare the stabilizing effect of Arg·Glu versus Arg·HCl and the effect of pH. The formulated mAbs were transferred to glass vials and stored for a number of weeks at controlled temperatures of 5, 25 and 40 °C (40 °C was considered to represent stress conditions). The samples were tested every week by SE-HPLC, using a Tosoh TSKgel column (with 5 μm beads) attached to Agilent HPLC 1200 system, monitored at 280 nm. Samples of 25 μl were injected each time, with a running buffer of 100 mM sodium phosphate, 100 mM NaCl, pH 6.8, and monitoring the absorbance profile at 280 nm. The peaks on the chromatograms were analysed to determine the percentage of mAb monomer present in solution, with each experiment carried out in duplicate.

## 3 Results

### 3.1 The effect of Arg·Glu on mAb colloidal stability

The *T*_agg_ value, derived from the temperature dependence of the SLS signal, was used as a measure to assess the colloidal stability of four mAbs tested, as a function of solution pH (in the range 5–7) and Arg·Glu concentration (0–200 mM). This pH range included the mildly acidic region (pH 5–6) commonly used for mAb formulation, and neutral pH which may sometimes be expected to promote mAb aggregation, due to its proximity to pI. Previous experimental studies have showed that whereas an addition of equimolar l-Arg and l-Glu at concentrations as low as 50 mM increases the solubility of proteins prone to aggregation in a concentration-dependent manner (Golovanov et al., 2004), more recent theoretical in silico studies suggested that l-Arg and l-Glu may reach a maximum effect at ∼150 mM, at least for a model protein used in simulations (Shukla and Trout, 2011). Therefore, the range of Arg·Glu concentrations chosen for the current study was extended up to 200 mM, which encompassed typical osmolalities used in the formulation of proteins for subcutaneous injection (Supporting Information, Fig. S1). In a control experiment, no significant light scattering was observed for Arg·Glu solutions in the absence of protein. The light scattering in the presence of protein in subsequent experiments therefore was directly attributed to protein aggregation (Supporting Information, Fig. S2).

**Fig. 1:**
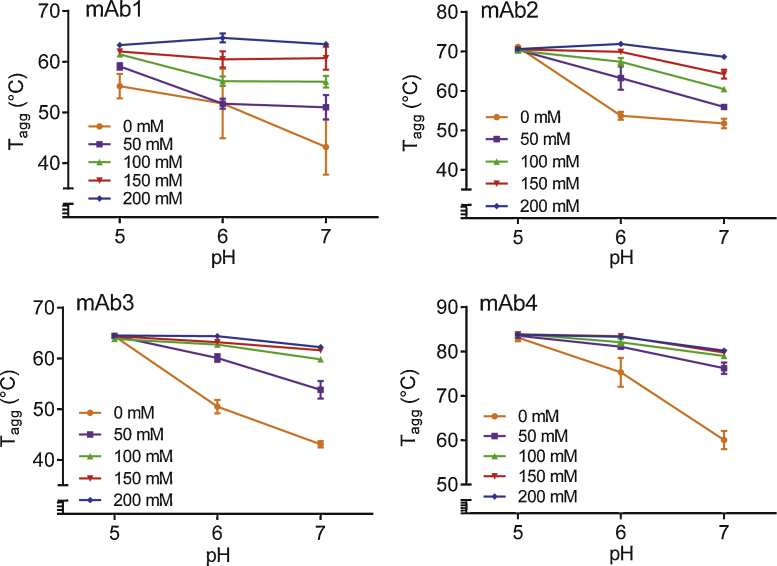
Change in *T*_agg_ as a function of pH for different concentrations of added Arg·Glu for the four mAbs as labelled. Error bars represent the standard deviation for three independent experiments.

**Fig. 2:**
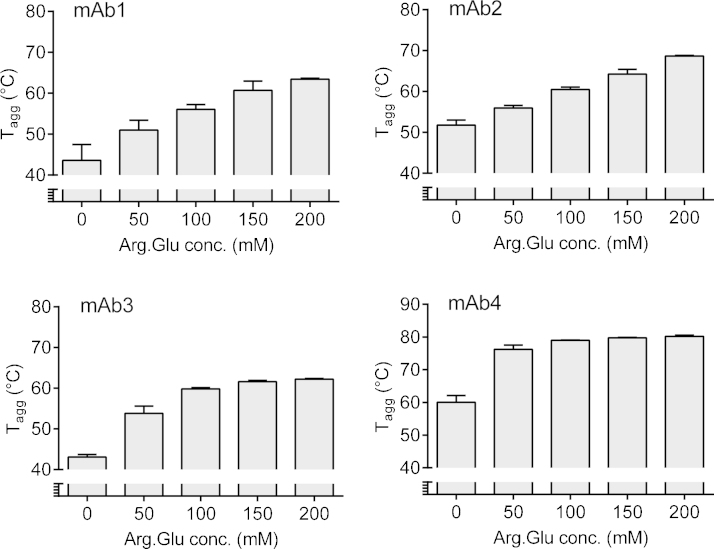
Change in *T*_agg_ of four mAbs at pH 7 as a function of Arg·Glu concentrations, as labelled. Error bars represent the standard deviation for three independent experiments.

The intrinsic *T*_agg_ values (i.e. those observed at pH 5, which is furthest away from the pI, and in the absence of Arg·Glu) for the four mAbs were quite diverse (ranging between ca. 54 and 83 °C), showing that the mAbs selected for these studies represent a wide range of inherent colloidal stabilities (Fig. 1). In the absence of Arg·Glu all the mAbs showed a consistent downward trend in *T*_agg_ values with increasing pH (Fig. 1). The addition of Arg·Glu however largely recovered the fall in *T*_agg_ in a concentration-dependent manner, such that for a maximal concentration of Arg·Glu at pH 7, the *T*_agg_ was very close to that at pH 5, and for mAb1 it was actually 20 °C higher (Fig. 1). At pH 5, the *T*_agg_ values for all mAbs except mAb1 were insensitive to the Arg·Glu concentration added. For mAb1 at pH 5, *T*_agg_ systematically increased with increasing Arg·Glu concentrations, by up to 9 °C. Moreover, increasing Arg·Glu concentrations increased *T*_agg_ for mAb1 (which had the lowest inherent *T*_agg_ of all antibodies tested here) consistently over all pH tested (Fig. 1). For all mAbs at pH > 5, the addition of Arg·Glu increased their colloidal stability, with this effect being more noticeable at higher, neutral pH. It is interesting that at neutral pH 7 (see Fig. 2) the *T*_agg_ continued to increase as Arg·Glu concentration was increased for mAb1 and mAb2, whereas for mAb3 and mAb4 the increase in *T*_agg_ reached a plateau at ca. 100 mM Arg·Glu and with the significant stabilizing effect being observed when adding just 50 mM Arg·Glu (Fig. 2).

### 3.2 The effect of Arg·Glu on mAb conformational stability

**Fig. 3:**
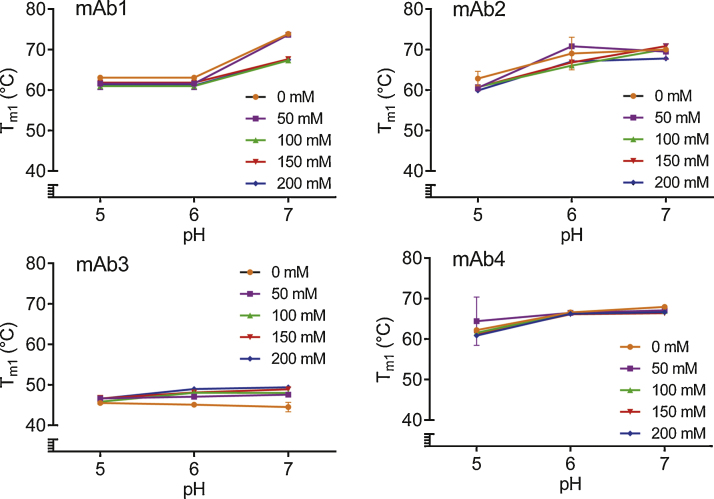
Change in *T*_m1_ as a function of pH for different concentrations of added Arg·Glu for the four mAbs as labelled. All graphs have the same *y*-axis scale to enable relative comparisons of thermal stabilities of different mAbs. Error bars represent the standard deviation for three independent experiments.

Conformational stability of mAbs was assessed here by the melting temperature *T*_m1_, measured from the temperature dependence of intrinsic fluorescence signal (a typical trace is shown in Supplementary Information, Fig. S3). In a control experiment, no fluorescent signal was detected in the absence of protein, for all Arg·Glu concentrations tested in C–P buffer at the different pH (data not shown), confirming that the intrinsic fluorescence measured was related to the protein sample only. As can be seen in Fig. 3, generally, an increase in solution pH caused increase in *T*_m1_ for most of the mAbs and most of the buffer conditions tested; the exception being a small decrease in *T*_m1_ in the buffer without Arg·Glu for the mAb3. Notably, for mAb1, mAb2 and mAb4, *T*_m1_ values were between 60 and 65 °C at pH 5 and rose towards ∼70 °C at pH 7, while T_m1_ values for mAb3 at pH 5 were ∼46 °C and rose towards ∼50 °C at pH 7 in the presence of Arg·Glu. The mAb3 is appreciably less conformationally stable compared to the other mAbs tested under these buffer conditions.

For all mAbs the general increase in *T*_m1_ with pH was relatively little influenced by the addition of Arg·Glu. However, for the least stable of the four antibodies, mAb3, a stabilizing effect of Arg·Glu was observed, especially at pH 7 where the difference in *T*_m1_ for 0 and 200 mM Arg·Glu was 5 °C (Fig. 3). No clear trend in *T*_m1_ versus Arg·Glu concentration was observed for mAb2, and for mAb1 and mAb4 a possible marginal destabilization at a given pH could be inferred (although this effect was only observed at pH 7 for mAb1 and was rather insignificant for mAb4, Fig. 3). Thus, the *T*_m1_ results indicated that Arg·Glu in the concentrations up to 200 mM used here, had a relatively small and variable, mAbs-dependent effect on conformational stability. However, most importantly, although the direct effect of Arg·Glu on the melting temperature *T*_m1_ was relatively small, the effect of raising the pH towards neutral had a more significant and consistent effect, raising the *T*_m1_ of all mAbs tested here. The conformational stability which can be achieved in the presence or absence of Arg·Glu as reflected by *T*_m1_, was always higher at pH 7 than at pH 5.

### 3.3 Controlled temperature storage stability studies

To investigate how the presence of Arg·Glu affected the overall long-term stability of mAbs formulated at higher concentrations, several test conditions (pH and additives) and three mAbs which show a characteristic behaviour were selected. We first explored the observed insensitivity of *T*_agg_ of mAb1 to pH in the presence of high concentration of Arg·Glu by formulating at pH 5.5 and 7.0, and second, compared the effect of adding Arg·HCl versus Arg·Glu on mAb3 (which is the least conformationally-stable) and mAb4 (which is comparatively stable). The resultant formulations are summarized in Table 1.

**Table 1:** Formulation conditions chosen for comparative accelerated stability studies of the three selected mAbs.

Base buffer	mAb1	mAb3	mAb4
	10 mM citrate–phosphate + 200 mM Arg·Glu	10 mM citrate–phosphate, pH 5.5	10 mM citrate–phosphate, pH 5.5
Formulation 1	pH 5.5	+200 mM Arg·Glu	+200 mM Arg·Glu
Formulation 2	pH 7.0	+200 mM Arg·HCl	+200 mM Arg·HCl

**Fig. 4:**
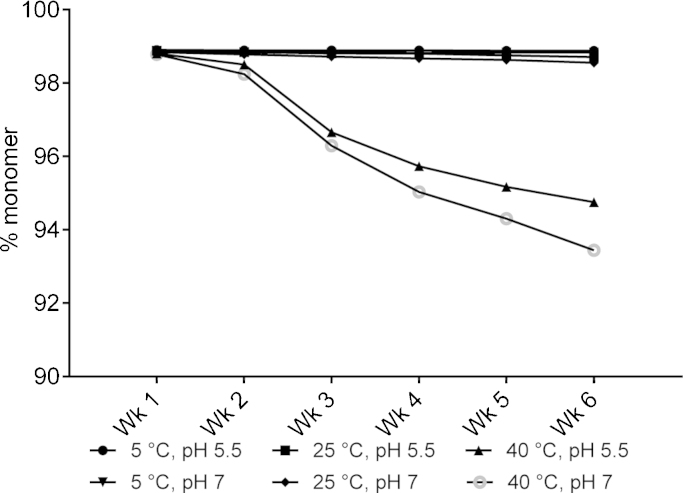
Monomer loss determined from SE-HPLC data for mAb1 stored over 6 weeks at 5, 25 and 40 °C in 10 mM CP buffer, 200 mM Arg·Glu pH 5.5 or 7, as labelled. The percent monomer at time = 0 for both formulations was 99.0%. Data reported were derived from two independent measurements which differed by <5%.

The solutions of chosen mAbs were prepared at 30–50 mg/ml concentration and taken forward to check the long-term stability of these samples after storage at different temperatures. The percentage of monomeric protein remaining in solution was monitored over time to assess the effect of condition parameters, in a comparative fashion. The colloidal stability of mAb1 in the absence of Arg·Glu was clearly better at low pH (Fig. 1): interestingly, in the accelerated stability study in the presence of Arg·Glu the difference in loss of monomer at pH 7.0 versus pH 5.5 was a relatively small value of <2% after storage at 40 °C for 6 weeks (Fig. 4). The higher inherent aggregation propensity at pH 7.0 for mAb1 can be explained by it being closer to its pI, measured to be in the region 7.9–8.3 (data not shown), which is the lowest pI of the four mAbs tested. In the absence of Arg·Glu the *T*_agg_ at this pH is only 43 °C (see Fig. 1) which is close to the storage temperature of 40 °C used in this experiment. It is rather remarkable then, that in the presence of 200 mM Arg·Glu mAb1 remains appreciably stable for weeks at 40 °C at pH 7, with more than 93% of monomeric form still remaining in solution after six weeks.

**Fig. 5:**
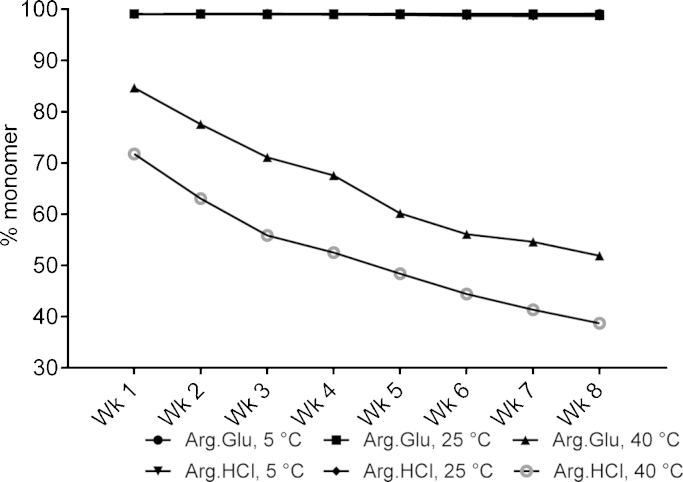
Monomer loss determined from SE-HPLC data for mAb3 stored over 8 weeks at 5, 25 and 40 °C in 10 mM CP buffer pH 5.5, with addition of arginine salts Arg·Glu or Arg·HCl, as labelled. The percent monomer at time = 0 for both formulations was 99.1%. Data reported were derived from two independent measurements which differed by <5%.

The mAb3 was the least conformationally stable antibody, with the lowest *T*_m1_ (Fig. 3). For mAb3, the performance of Arg·Glu versus Arg·HCl as an enhancer of the long-term stability was compared at pH 5.5 (see Table 1). Under accelerated conditions at 40 °C, it became possible to discern a clear preference for Arg·Glu over Arg·HCl over all time points tested (Fig. 5). Already after one week at 40 °C, there was a dramatic decline of almost 30% in the monomer population in the presence of Arg·HCl, which is halved in the presence of Arg·Glu (∼15% monomer loss). Similarly to what was observed for mAb1, there were only small (<5%) differences in mAb3 stability when stored at 5 or 25 °C. Much longer storage times, up to several months, would probably be required to distinguish between the two arginine salts at these lower temperatures. It should be noted that at 40 °C, mAb3 was not far from its intrinsic *T*_m1_ (45 °C, see Fig. 3) which would explain the relatively fast decrease in the monomer population observed for this mAb3 when stored at this higher temperature. Overall, for mAb3 Arg·Glu provided a better storage stabilization than Arg·HCl. As a combination of pH 7 with 200 mM Arg·Glu increases *T*_m1_ for mAb3 by about 5 °C (see Fig. 3) without significantly compromising *T*_agg_ (Fig. 1), it will be interesting to check in the future if using pH 7 would afford a better overall storage stability of this antibody at high temperature.

**Fig. 6:**
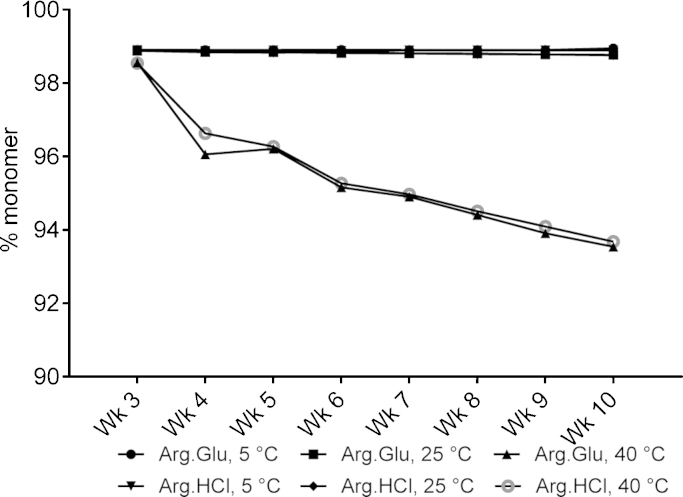
Monomer loss determined from SE-HPLC data for mAb4 stored over 10 weeks at 5, 25 and 40 °C in 10 mM CP buffer pH 5.5, with addition of arginine salts Arg·Glu or Arg·HCl, as labelled. The percent monomer at time = 0 for both formulations was 98.9%. Data reported were derived from two independent measurements which differed by <5%. Only data from week 3 onwards is shown on the graph as there was no significant change in the percent monomer before that (data for weeks 1 and 2 are very similar to week 3).

The third antibody tested, mAb4, has relatively high values of both *T*_agg_ and *T*_m1_ (see Fig. 1, Fig. 3), and hence is a comparatively stable antibody. Here, we compared the relative performance of Arg·Glu versus Arg·HCl. Despite extending storage for up to 10 weeks, no obvious difference in monomer loss was observed for addition of 200 mM Arg·Glu or Arg·HCl, at all temperatures (5, 25 and 40 °C) (Fig. 6). Similarly to observations for mAb1 and mAb3, there was less than 1% loss of monomer over the study period at 5 or 25 °C, while larger losses were recorded at 40 °C; although in the case of mAb4 these losses were small at ∼4% over 6 weeks. For mAb4 formulated at pH 5.5, we have not found significant difference between performance of Arg·Glu versus Arg·HCl (Fig. 6). Interestingly however, the mAb1, which is inherently prone to aggregation, when formulated in 200 mM Arg·Glu showed a monomer decay pattern (Fig. 4) very similar to that of a ‘stable’ mAb4 (Fig. 6), suggesting that usage of Arg·Glu in formulations can be beneficial, especially for mAbs which are less stable and more prone to aggregation. Although for mAb4, which was stable under the stress conditions tested in this study, no immediate further benefit of using Arg·Glu versus Arg·HCl was observed, it still remains to be explored if this excipient can have beneficial effect on stability under more extreme stress conditions, when even the most stable mAbs start to degrade, e.g. during long-term room-temperature storage.

## 4 Discussion

The main purpose of this study was to examine the concentration-dependent effect of Arg·Glu on the physical stability of representative mAbs at different pH. Either (or both) colloidal and conformational stability can control the rate and extent of aggregation, which is one of the main degradation pathways for therapeutic mAbs (Goldberg et al., 2011). The colloidal stability, or the ability of solution conditions to reduce self-association and aggregation of mAbs can be conveniently assessed by recording the temperature, *T*_agg_, at which SLS signal, which is sensitive to formation of aggregates in solution, starts to increase (Avacta, 2013b, Goldberg et al., 2011). The conformational stability, or the ability of protein to maintain its native folded state, can be assessed in different formulation conditions by looking at the temperature of the first unfolding transition, *T*_m1_, measured using temperature dependence of intrinsic fluorescence signal (Avacta, 2013b, Goldberg et al., 2011). These methods require only small amounts of samples and allow quick and high-throughput assessment of whether protein is stabilized or destabilized under particular solution conditions (Avacta, 2013b, Goldberg et al., 2011).

Initial investigation of the colloidal stability of the four mAbs in the pH range 5–7 revealed a general drop in the *T*_agg_ as pH came closer to pI, however this drop was largely recovered upon the addition of Arg·Glu. In the case of mAb3 and mAb4, 50 mM Arg·Glu was sufficient to bring about a near-complete recovery of the *T*_agg_, corroborating earlier findings showing the same concentration significantly increased protein solubility (Golovanov et al., 2004). For the same mAbs increasing Arg·Glu concentrations above 100 mM yielded almost no further increase in *T*_agg_, suggesting that an optimal concentration of these additives may indeed exist, which is in agreement with theoretical simulations (Shukla and Trout, 2011). Such concentration may be in the range of osmolalities which are pharmaceutically-acceptable for the antibody formulations intended for injections. In the case of mAb1 and mAb2, the Arg·Glu concentration-dependent increase in *T*_agg_ did not reach saturation at pH 6–7, therefore the optimal concentration of Arg·Glu may be different for different mAbs and concentrations above 200 mM may be beneficial in some cases for a greater stabilization against aggregation. Interestingly, for the majority of mAbs tested here, the recovery in *T*_agg_ by Arg·Glu appeared to be limited to a *T*_agg_ value observed at pH 5, i.e. for mAb1 and mAb2 it is likely that a saturable limit also exists above 200 mM Arg·Glu.

Generally, at pH 5, the effect of addition of Arg·Glu on *T*_agg_ was significant only for the most aggregation-prone antibody, mAb1, which had the lowest *T*_agg_. This addition increased the value of *T*_agg_ by up to 9 °C, to the typical level observed for other, more stable antibodies (Fig. 1). However, the *T*_agg_ values of antibodies which were already stable against aggregation at this pH were not affected. The likely explanation for this is that the colloidal stability of these proteins is already enforced by the electrostatic repulsion of the positively-charged protein molecules, as pH is far away from the pI.

Consideration of conformational stability is important in formulation since occurrences of partial protein unfolding may lead to the exposure of hydrophobic ‘sticky’ patches and subsequent aggregation. Ideally, conformational stability should be maximized. The intrinsic fluorescence data during thermal denaturation for all the mAbs shows a general increase in *T*_m1_ as pH increased from 5 to 7. In comparison with this general trend, the addition of Arg·Glu had relatively small and variable direct effect on the *T*_m1_ of the mAbs studied, although mAb3, the least conformationally-stable antibody, presented an interesting case since its conformational stability increased with Arg·Glu addition in a concentration-dependent manner. A molecular mechanism for this effect may relate to the ion-specific effects of Arg·Glu via the guanidinium group, which is chaotropic in nature within the Hofmeister series. Collins (1997) proposed that chaotropic ions specifically bind like-charged chaotropic residues (i.e. arginine, lysine) on the protein surface (and vice versa for kosmotropes) (Collins, 1997). Further experiments would be required, comparing the effects of Arg·Glu and Arg·HCl on mAb3, especially at pH 7, to clarify the mechanism of its structural stabilization. Overall, from the conformational stability perspective, there appears to be a clear advantage in using pH closer to 7 for all four mAbs tested here.

Given that the addition of Arg·Glu at pH 6–7 enables to largely recover the *T*_agg_ back to the values typically observed at pH 5, it may be interesting to exploit this effect in the context of hypothetical formulations for mildly basic proteins at a pH around neutral. Generally, protein therapeutics are formulated so that the pH of the formulation is at least 1 unit away from the pI of the molecule (Lehermayr et al., 2011). In theory however, when the solution pH is equal to the protein's pI the electrostatic contributions to conformational stability should be maximal (Ugwu and Apte, 2004). Our observations for *T*_m1_ dependence on pH (Fig. 3) fully support this prediction experimentally. It follows that if Arg·Glu can indeed mitigate the loss of colloidal stability on moving towards the pI of a mildly basic protein, there may exist the possibility of increasing the formulation space towards higher pH values, where protein is inherently more stable conformationally. This possibility has been previously postulated in the context of formulation in the biopharmaceutical industry (Banga, 2006).

The *T*_agg_ and *T*_m1_ data demonstrate that, in the range of concentrations giving rise to clinically-acceptable solution osmolality, Arg·Glu had a significant, positive direct effect on the mAbs' colloidal stability but a relatively small direct effect on their conformational stability. These findings agree with previous studies which showed that Arg·Glu appears to attenuate protein self-association and does not significantly affect the conformation of natively folded protein (Golovanov et al., 2004, Vedadi et al., 2006). Using molecular dynamics, Shukla and Trout (2011) proposed a molecular mechanism wherein hydrogen bonding between Arg and Glu facilitates an increase in the concentration of both ions at the protein surface, augmenting the crowding effect and suppressing protein–protein interaction (Shukla and Trout, 2011). Interestingly, recently it was suggested that at high concentrations (0.5 M) the presence of l-Glu or l-Asp as counter-ions for l-Arg may mitigate some of the destabilizing effects of l-Arg alone (Fukuda et al., 2014).

In addition to investigating the effect of Arg·Glu on *T*_agg_ and *T*_m1_ individually when mAbs are at low concentrations, it is important to check whether any perceived improvements in these stability parameters translate into improvements in the overall storage stability in more concentrated form, especially at elevated temperature. We selected a set of formulation conditions and three antibodies showing characteristic inherent behaviour: one with low *T*_agg_ (mAb1), one with low *T*_m1_ (mAb3), and one comparatively stable (mAb4). We explored the long-term storage stability (i.e. the percentage of monomer still remaining in the solution) of these antibodies in the presence of 200 mM Arg·Glu comparing it with a reference formulation. Although the rate of aggregation at 40 °C of mAb1 was slightly less (by <2%) at pH 5.5 than at pH 7, the overall storage stabilization afforded by Arg·Glu at neutral pH for this protein at higher concentration is itself remarkable. Indeed, in the absence of Arg·Glu at pH 7 which is approaching the pI, mAb1 even at low concentration is heavily prone to aggregation at elevated temperature. The assessment of colloidal and conformational stability of this mAb1 performed at lower protein concentration (Fig. 1) has revealed that in the presence of 200 mM Arg·Glu this protein becomes colloidally-stable over the whole range of pH. The long-time storage experiment for mAb1 under thermal stress thus confirmed that the overall stability of this formulation at pH 7 indeed is very similar to that at pH 5.5, which highlights the usefulness of assessing *T*_agg_ and *T*_m1_ as a way to predict the overall protein stability in different conditions.

Another protein, mAb3, which had the lowest inherent *T*_m1_, also demonstrated an interesting behaviour: at pH 5.5 the suppression of aggregation was significantly greater for Arg·Glu than for Arg·HCl. Despite the proximity of its inherent *T*_m1_ value of 45 °C to the storage temperature, in the presence of 200 mM Arg·Glu a significant proportion of this protein (52%) remained in solution as monomer even after 8 weeks. Given that the inherent *T*_m1_ of mAb3 at pH 7 and in the presence of Arg·Glu is expected to be higher than at pH 5.5 used in this experiment (see Fig. 3), it would be interesting in the future to assess its long-term storage stability under thermal stress at pH 7, and compare that with the performance of another l-Arg salt, Arg·HCl. The benefit of using Arg·Glu versus Arg·HCl was not however evident for the most stable mAb4: more challenging stress conditions may be needed for such discrimination in this case. Overall, these data support our proposal (based on *T*_agg_ and *T*_m1_ data) that the use of Arg·Glu may facilitate the formulation of mildly basic proteins at neutral pH. The relative advantages of using Arg·Glu as opposed to Arg·HCl (albeit, at much higher concentrations of these amino acids than used in the current study) were also recently highlighted by Fukuda et al. (2014), suggesting that the presence of l-Glu or l-Asp as a counter-ion for l-Arg mitigate some of the destabilizing properties of arginine alone (Fukuda et al., 2014).

## 5 Conclusions

Arg·Glu has the potential to increase the physical stability of mAbs formulated at weakly acidic to neutral pH. Depending on the particular mAb, stabilization as measured by *T*_agg_ scales with increasing concentrations of Arg·Glu to at least 200 mM, or reaches a plateau around 100 mM with even 50 mM affording significant benefit. Thus, Arg·Glu is effective at relatively modest concentrations (<200 mM) giving rise to osmolalities acceptable for injections. The direct effect of Arg·Glu at these concentrations on the melting temperature *T*_m1_ of the mAbs appeared marginal, unlike the effect of pH itself, which significantly increases *T*_m1_ as pH approaches 7. Under accelerated stability conditions, Arg·Glu reduced monomer loss for the least stable mAb to a greater extent than observed for Arg·HCl, and suppressed aggregation at pH 7.0 to near that observed at pH 5.5. Arg·Glu may therefore be a potential alternative to Arg·HCl in mAb formulation development, though further studies will be required to better understand its applicability at higher protein concentrations, by assessing a wider range of formulation conditions.
