# Supplementary material for: The effect of arginine glutamate on the stability of monoclonal antibodies in solution
Source: Int J Pharm. 2014 Oct 1;473(1-2):126–33. doi: 10.1016/j.ijpharm.2014.06.053 (PMC4162492; doi:10.1016/j.ijpharm.2014.06.053)
Supplement: Supplementary file 1 [file mmc1.pdf]

Supplementary Information

The effect of arginine glutamate on the stability of monoclonal antibodies in solution

Priscilla Kheddo,[a,b] Malgorzata Tracka,[c] Jonathan Armer,[c] Rebecca J. Dearman,[b] Shahid Uddin,[c] Christopher F. van der Walle,[c] Alexander P. Golovanov [a,b,]*

[a] Manchester Institute of Biotechnology, University of Manchester, Manchester, M1 7DN, UK

[b] Faculty of Life Sciences, University of Manchester, Manchester, M13 9PL, UK

[c] MedImmune Ltd, Granta Park, Cambridge, CB21 6GH, UK

* Corresponding author at: Manchester Institute of Biotechnology and Faculty of Life Sciences, The University of Manchester, 131 Princess Street, Manchester M1 7DN, UK. Tel.: +44 161 306 5813; Fax: +44 161 306 5201

Email address: A.Golovanov@manchester.ac.uk (A.P. Golovanov)

Supplementary Figures

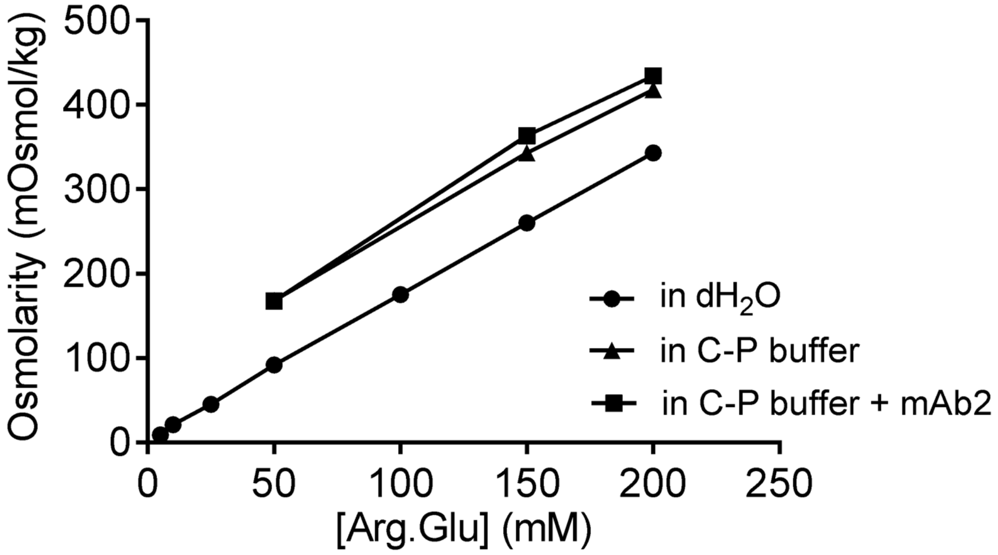

Fig. S1. Osmolality of Arg•Glu solutions prepared in distilled water, 10mM citrate-phosphate (C-P) buffer pH 6.0, and 10 mM C-P buffer containing 30 mg/ml mAb2, as indicated. Duplicate measurements of independent samples were plotted and the variation between measurements was < 5%.

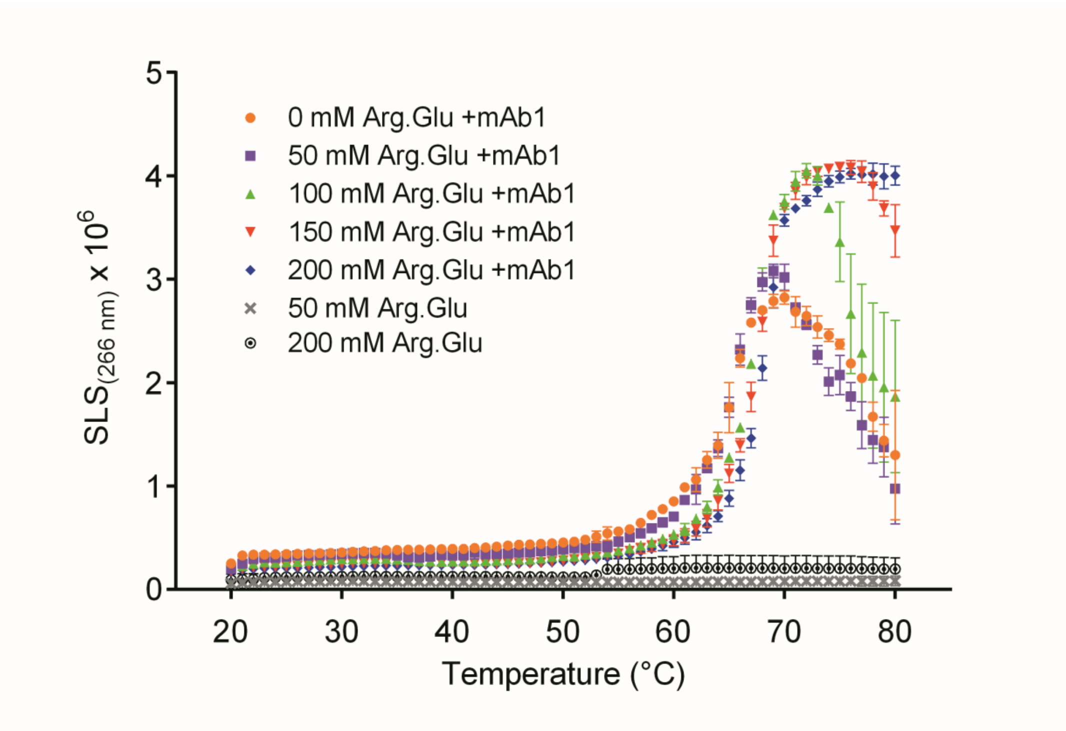

Fig. S2. Example data showing the SLS signal at 266 nm used to measure T_{agg}, here for mAb1 in C-P buffer, pH 5 (*cf*. values in Fig. 1, main text). The amount of light scatter for Arg•Glu solutions in C-P buffer pH 5 without mAb1 was relatively insignificant, such that the SLS signal for Arg•Glu solutions containing mAb1 represent light scatter from mAb aggregation. The decrease in SLS signal at temperatures above ~ 70 °C for mAb1 samples was due to the gross precipitation of the protein sample and consequent light obscuration. Similar traces were obtained for the other mAbs and different pH citrate-phosphate buffer. Data were analyzed using GraphPad Prism v6, plotting averages of measurements from three independently prepared samples with error bars representing the standard deviation.

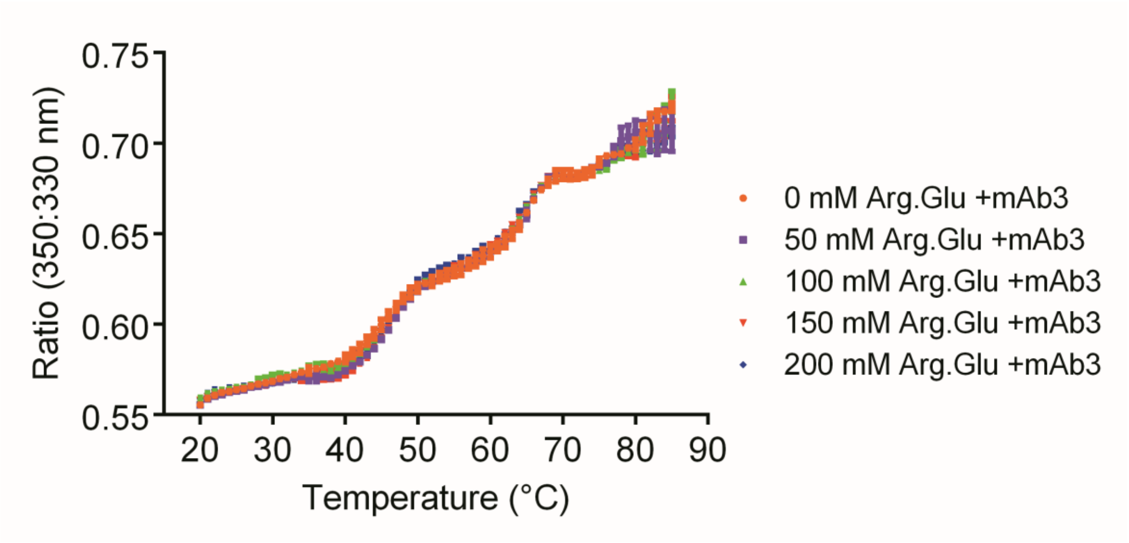

Fig. S3. Example intrinsic fluorescence data showing the ratio of the intensities at 350 and 330 nm, used to measure T_{m1}, here for mAb3 in C-P buffer, pH 5 (*cf*. values in Fig. 3, main text). The first unfolding transition is most clearly seen and occurs between *ca*. 40 and 50 °C, corresponding to the approximate locations of the lower and upper baselines respectively, giving T_{m1} value of ~45 °C. Similar traces were obtained for the others mAbs and different pH C-P buffers. Data were analyzed using the Avacta Optim 2 software and GraphPad Prism v6, plotting averages of measurements from three independently prepared samples with error bars representing the standard deviation.
